# Modulation of Type I Interferon Responses to Influence Tumor-Immune Cross Talk in PDAC

**DOI:** 10.3389/fcell.2022.816517

**Published:** 2022-02-22

**Authors:** Carlotta Cattolico, Peter Bailey, Simon T. Barry

**Affiliations:** ^1^ Bioscience, Early Oncology, AstraZeneca, Cambridge, United Kingdom; ^2^ Institute of Cancer Sciences, University of Glasgow, Glasgow, United Kingdom; ^3^ Department of Surgery, University of Heidelberg, Heidelberg, Germany; ^4^ Section Surgical Research, University Clinic Heidelberg, Heidelberg, Germany

**Keywords:** innate immunity, PDAC, IFN-I, nucleic acid sensing, immunotherapy, tumor microenvironment

## Abstract

Immunotherapy has revolutionized the treatment of many cancer types. However, pancreatic ductal adenocarcinomas (PDACs) exhibit poor responses to immune checkpoint inhibitors with immunotherapy-based trials not generating convincing clinical activity. PDAC tumors often have low infiltration of tumor CD8^+^ T cells and a highly immunosuppressive microenvironment. These features classify PDAC as immunologically “cold.” However, the presence of tumor T cells is a favorable prognostic feature in PDAC. Intrinsic tumor cell properties govern interactions with the immune system. Alterations in tumor DNA such as genomic instability, high tumor mutation burden, and/or defects in DNA damage repair are associated with responses to both immunotherapy and chemotherapy. Cytotoxic or metabolic stress produced by radiation and/or chemotherapy can act as potent immune triggers and prime immune responses. Damage- or stress-mediated activation of nucleic acid-sensing pathways triggers type I interferon (IFN-I) responses that activate innate immune cells and natural killer cells, promote maturation of dendritic cells, and stimulate adaptive immunity. While PDAC exhibits intrinsic features that have the potential to engage immune cells, particularly following chemotherapy, these immune-sensing mechanisms are ineffective. Understanding where defects in innate immune triggers render the PDAC tumor–immune interface less effective, or how T-cell function is suppressed will help develop more effective treatments and harness the immune system for durable outcomes. This review will focus on the pivotal role played by IFN-I in promoting tumor cell–immune cell cross talk in PDAC. We will discuss how PDAC tumor cells bypass IFN-I signaling pathways and explore how these pathways can be co-opted or re-engaged to enhance the therapeutic outcome.

## Introduction

PDAC accounts for more than 90% of pancreatic malignancies and is currently the third leading cause of cancer-related death in Western countries (Luchini et al., 2016; Pishvaian and Brody, 2017; McGuigan et al., 2018). High mortality rates are mainly due to late detection, a high level of tumor heterogeneity, and a desmoplastic immunosuppressive microenvironment (Ryan et al., 2014). Although advances in treatment have significantly increased 5-year survival rates for many other cancer types (Brahmer et al., 2012; Nixon et al., 2018), the overall 5-year survival rate for PDAC is only 10% (Siegel et al., 2021). Mono- and multi-agent chemotherapy regimens are the standard treatments. Conventional cytotoxic therapies in PDAC include the nucleoside analog gemcitabine which may be combined with the microtubule poison nab-paclitaxel, and FOLFIRINOX (folinic acid, fluorouracil, irinotecan, and oxaliplatin) (Mohammad, 2018). Both treatment regimens improve the overall survival for patients with localized disease but are less effective for the majority of patients who are diagnosed with advanced or metastatic disease (Bliss et al., 2014). Improvements in progression-free survival have been achieved for patients with BRCA-mutated tumors when PARP inhibitors are used as maintenance treatment following chemotherapy (Golan et al., 2019). Therefore, despite incremental advances, there is an urgent need for new therapeutic approaches.

Immune checkpoint inhibitors (ICIs) are revolutionizing the treatment of cancer, mainly when used in combination with chemotherapy or radiotherapy. In ICI-sensitive diseases such as non-small-cell lung cancer (Lim et al., 2020), head and neck squamous cell carcinoma (Burtness et al., 2018), and triple-negative breast cancer (Schmid et al., 2018), combining chemotherapy and immunotherapy generates durable responses in a subset of patients (Reck, 2018). Combining standard-of-care chemotherapy with ICIs has been largely unsuccessful in PDACs [reviewed in Henriksen et al. (2019)], albeit in trials where patients were not selected based on any favorable biomarker.

PDAC is generally considered immunologically cold with a low incidence of tumor-infiltrating lymphocytes (TILs) (Stromnes et al., 2017; Blando et al., 2019; Gorchs et al., 2019; Seo et al., 2019), thought to be a result of poor T-priming by the tumor. Poor T-cell priming or activation in PDAC is commonly attributed to low neo-antigen content, although PDAC tumors do have potentially actionable neo-epitopes. Indeed PDAC tumors with good quality neo-antigens are associated with better outcomes (Bailey et al., 2016a; Balachandran et al., 2017). How PDAC tumor cells evade the immune system is unclear. Tumor cell intrinsic mechanisms play important roles in regulating both intrinsic and therapy-induced engagement or avoidance of the innate or adaptive immune systems (reviewed in Wellenstein and de Visser (2018)). Tumor cells can evade the immune system by reducing antigen processing, cell surface antigen presentation, or expression of cell surface proteins that engage innate immune cells. They also secrete growth factors, chemokines, and cytokines that shape an immunosuppressive microenvironment (Grivennikov et al., 2010; Gonzalez et al., 2018). In addition to high tumor T-cell infiltration, “immunologically hot” tumors that respond well to immunotherapy-based treatment typically express IFN-I (IFN-α, -β), type II interferons (IFN-II) (IFN-γ), and high levels of interferon-stimulated genes (ISGs) that sustain antitumor immune responses (Gajewski et al., 2013; Corrales et al., 2015; Wang and Wang, 2017). PDAC does not commonly show high expression of interferons or ISG signatures, but if tumor-targeted therapies such as chemotherapy or targeted agents induce robust IFN-I and/or innate responses, immune system engagement is more likely to occur. Understanding why this fails in PDAC could guide new therapeutic or patient selection approaches that will ultimately harness the immune system to deliver more durable clinical responses.

## IFN Cascade in Tumors

**FIGURE 1:**
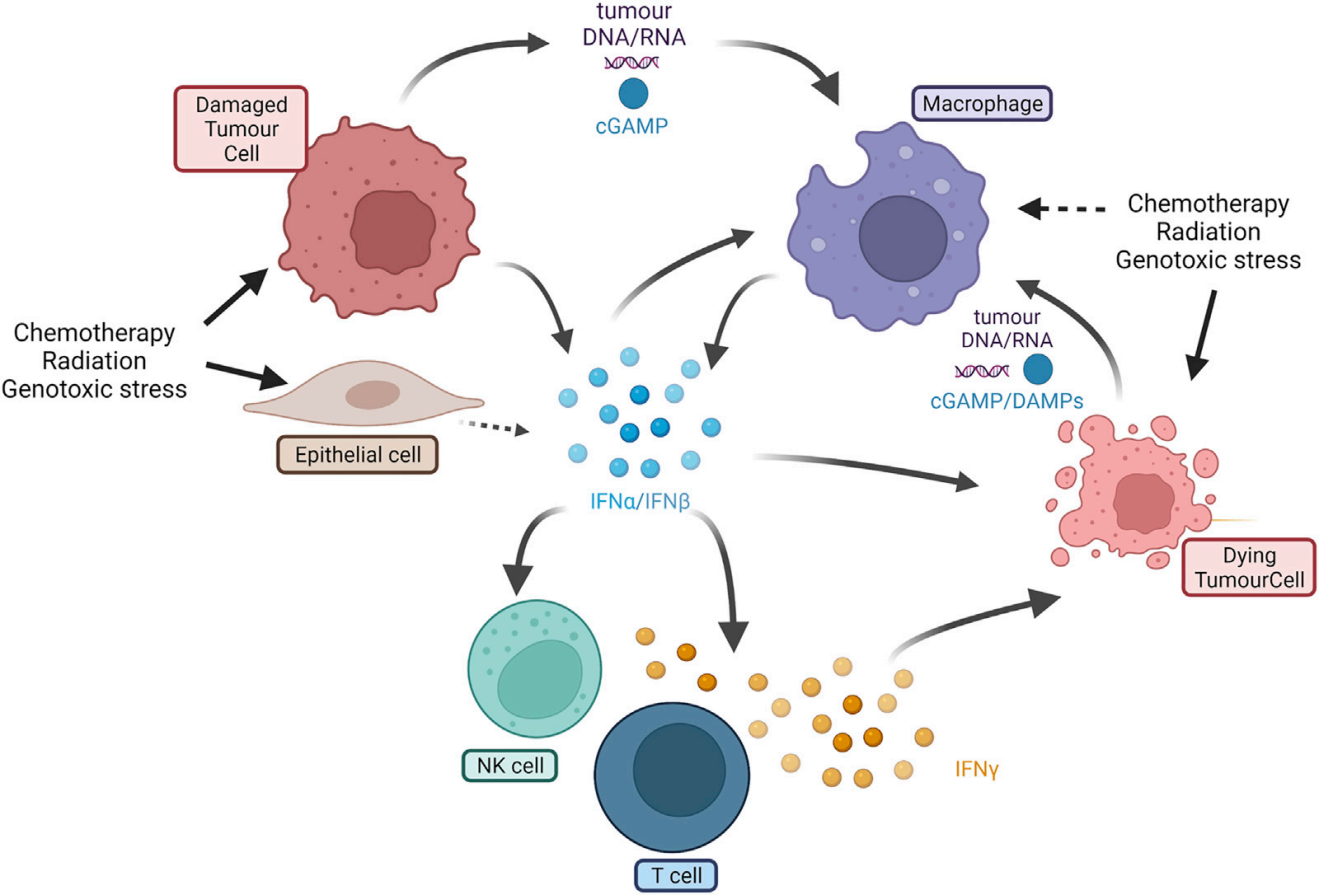
Overview of IFN-I and IFN-II cascade in tumors. IFN-I (interferons *α* and β) can be expressed by damaged tumor cells, epithelial and stromal cells (e.g., macrophages) as a result of cell damage or stimulation. Secreted IFN-I activates macrophages, NK cells, and T cells, and kills other tumor cells. Cross talk can also be mediated by DNA or RNA fragments or DAMPs such as cGAMP. T cells produce IFN-II (IFN-γ) that is cytotoxic to tumor cells. Dying tumor cells can release further DAMPs or cell fragments that further stimulate new macrophages or other innate immune cells (e.g., dendritic cells not shown). IFN = interferon; NK = natural killer; cGAMP = cyclic guanosine monophosphate; DAMP= damage-associated molecular patterns (Created using BioRender®).

The IFN cascade in tumors is complex, as shown in Figure 1. Type I interferons (IFN-α and -β) can be expressed as a result of stress damage to tumor cells or stromal cells such as macrophages (Medrano et al., 2017). Type I interferons stimulate many cells in the TME driving both tumor cell and immune cell responses (Zitvogel et al., 2015). The secretion of type I interferons can kill or senesce tumor cells and directly or indirectly stimulate T cells, natural killer (NK) cells (Fenton et al., 2021), and potentially macrophages or dendritic cells to secrete the type II interferon (IFN-γ). The type II interferon is one marker of cytotoxic immune effector cells in the TME (Bhat et al., 2017). The IFN system can be self-enhancing. For example, chemotherapy-mediated damage may induce the secretion of IFNs from both tumor cells and cells in the TME amplifying immune cell activation throughout the tumor (Budhwani et al., 2018). Understanding the cause and effect in this pivotal cascade is challenging as both type I and -II interferons drive common signaling cascades. IFN responses in tumors are commonly monitored using bulk RNA gene expression signatures which, in the absence of single-cell sequencing approaches, are unable to distinguish which cells initiate, or which cells respond to IFN-driven signals. This complex interplay between cells in tumors presents different opportunities to drive IFN induction and activation of antitumor immune cell activity, even when normal pathways are dysfunctional.

## Chemotherapy and Immunomodulation in PDAC

Chemotherapeutic agents are first-line standard-of-care treatments for PDAC. Despite that the combination of gemcitabine with nab-paclitaxel and FOLFIRINOX increases efficacy compared to gemcitabine alone, patients’ overall survival rates remain particularly low, and toxicity limits their use (Conroy et al., 2011; Von Hoff et al., 2013; Javed et al., 2019). PDAC patients rapidly develop resistance to chemotherapy through both tumor cell intrinsic and tumor microenvironmental factors (Chand et al., 2016). Despite the limited benefit, there is evidence of chemosensitivity in subsets of PDAC patients. For instance, several clinical trials report increased sensitivity to platinum-based chemotherapy in DNA damage repair (DDR)-deficient patients (Pishvaian et al., 2019; Golan et al., 2014; O'Reilly et al., 2020; Blair et al., 2018).

In addition to tumor cell cytotoxic activity, chemotherapies may also potentiate immunomodulatory responses (Bailly et al., 2020; Piadel et al., 2020). Treatment of PDAC with gemcitabine has been associated with enhanced T-cell-mediated responses through increased naive T-cell activation (Plate et al., 2005) and CD8^+^ T-cell function, which is in part dependent on dendritic cell (DC) activity (Plate and Harris, 2004). In addition, following gemcitabine or FOLFIRINOX treatment, suppressive immune populations such as regulatory T cells (Tregs) (Kan et al., 2012; Homma et al., 2014; Liu et al., 2017; Hui et al., 2021; Sams et al., 2021) and myeloid-derived suppressor cells (MDSCs) (Suzuki et al., 2005; Vincent et al., 2010; Eriksson et al., 2016; Wu et al., 2020a) are downregulated in patients and in preclinical models. In mice, gemcitabine also increases the immunogenicity of tumor cells (Liu et al., 2010). Therefore, global changes in the immune profile of tumors following treatment can be both direct and indirect. Consistent with these findings, an *in vitro* analysis of the proteasome and immunopeptidome of human PDAC cell lines showed that gemcitabine induces overexpression of MHC-I molecules (Gravett et al., 2018) with novel peptides. Similarly, in KPC tumor-bearing mice, prolonged treatment with gemcitabine leads to increased MHC-I along with increased secretion of CCL/CXCL chemokines (Principe et al., 2020). Gemcitabine and oxaliplatin (the platinum-based element of the FOLFIRINOX regimen) also induce the expression of immunogenic cell death-associated damage-associated molecular patterns (DAMPs) including ATP and HMGB1 (Hayashi et al., 2020; Smith et al., 2021), which can signal NK cells and potentiate the innate immune system. Finally, in PDAC patients, chemotherapy has been shown to stimulate the immune response by increasing T-cell response to tumor-associated antigens (Mandili et al., 2020).

The immunostimulatory effects of these chemotherapeutics highlight their potential use in combination with immuno-oncology therapies. Especially with ICIs, *in vitro* and *in vivo* studies suggest that gemcitabine increases the expression of immune checkpoint molecules such as PD-L1 (Principe et al., 2020; Smith et al., 2021). While favorable changes in the immune profile of tumors can be seen following treatment, why these changes do not translate into more sustained benefit alone or in combination with other therapies remains unclear. This may be because the impact of chemotherapy on tumor cells is rapidly reversed or because the TME remodels quickly restraining the potential benefits. Understanding the kinetics of induction and recovery in the tumor cells versus TME following chemotherapy treatment would give useful insights.

## Treatments Targeting Immune Cells Alone Are Not Active in PDAC

Clinically, antitumor immune responses are stimulated by therapeutic antibodies that target T-cell checkpoints such as PD-1/PD-L1 and, to a lesser extent, CTLA-4/B7H4, which act to inhibit T-cell function. In PDAC, both PD-L1 and CTLA-4 are upregulated and associated with poor prognosis (Nomi et al., 2007; Cancer Genome Atlas Resea, 2017). Early trials showed no beneficial response to ipilimumab (anti-CTLA-4 monoclonal antibody) in patients with metastatic and locally advanced PDAC (NCT00112580), although treatment was associated with advanced toxicity (Royal et al., 2010). These results were mirrored with another anti-CTLA-4 monoclonal antibody (NCT02527434), tremelimumab (Sharma et al., 2018). Anti-PD-L1 monoclonal antibodies showed no objective response in the 14 PDAC patients in a phase I clinical trial (NCT00729664) (Brahmer et al., 2012), while disease control (partial response or stable disease) was observed in 21% of the 29 PDAC patients in another phase I trial with durvalumab (NCT01693562) (Segal et al., 2014). Interestingly, PD-1 blockade with pembrolizumab showed a 62% objective response in a phase II clinical trial in 8 PDAC patients harboring mismatch repair (MMR) deficiency (NCT01876511) (Le et al., 2015). Pembrolizumab has now been approved by the Food and Drug Administration (FDA) for solid tumors harboring MMR defects including PDAC (Le et al., 2017); however, these only represent about 1% of PDAC patients (Hu et al., 2018). In the other patients, very limited responses to ICIs have been observed. Some studies are still ongoing such as a phase II trial of atezolizumab (NCT03829501), as well as combinations of antibodies targeting CTLA-4 and PD1/PD-L1 (NCT01928394). Consistent with findings in other tumor types, it is probable that combination approaches, for example, with chemotherapy, will be required.

Cell-based vaccines alone or combined with chemotherapy and ICI present alternative strategies to achieve tumor-targeted immune stimulation. The granulocyte–macrophage colony-stimulating factor (GM-CSF)-secreting allogeneic pancreatic tumor cell vaccine (GVAX) consists of irradiated human allogeneic pancreatic tumor cells that secrete GM-CSF (Lutz et al., 2011). It is given as a cellular vaccine to present pancreatic cancer cell epitopes and also improve antigen presentation by inducing GM-CSF-mediated maturation of dendritic cells. Despite showing positive changes in the immune microenvironment and potential efficacy in early clinical trials (Lutz et al., 2014), a more extensive phase II trial in combination with the CTLA4 targeting ICI ipilimumab failed to show increased clinical benefit (Wu et al., 2020b). Studies like this which assess immune biomarker changes suggest that while the activation of immune cells can be achieved in PDAC, they do not translate into a durable effect. The use of GM-CSF in GVAX is interesting as it has both positive and negative effects. In addition to promoting dendritic cell recruitment, increased GM-CSF can promote pancreatic tumor development (Pylayeva-Gupta et al., 2012) and drive recruitment of immunosuppressive immune cells (Bayne et al., 2012).

## Chemotherapy and Immunotherapy Combinations in PDAC

Greatest response to ICI treatment is observed in combination with chemotherapy. Both chemotherapy and radiotherapy can increase antitumor immunity by inducing immunogenic tumor cell death and cell stress signaling pathways, and depleting immunosuppressive cells or stimulation of T cells to complement the effects of ICI treatment (Burtness et al., 2018; Galluzzi et al., 2020; Salas-Benito et al., 2021). In PDAC, the combination of ICI with chemotherapy is marginally more effective than monotherapy ICI treatment; however, benefits remain limited. Combining ipilimumab with gemcitabine in a phase Ib trial (NCT01473940) resulted in 2 partial responses and five stable diseases out of 11 patients (Kamath et al., 2020). In a larger phase I clinical trial that combined tremelimumab and gemcitabine (NCT00556023), 2 out of 28 PDAC patients showed a partial response and seven stable diseases (Aglietta et al., 2014). Chemotherapy in combinations with ICIs compared to chemotherapy alone conferred longer overall survival in a clinical trial comprising 58 patients with advanced PDAC (Ma et al., 2020). Other phase I and Ib trials have combined nivolumab or pembrolizumab with nab-paclitaxel and gemcitabine. In the first of these trials (NCT02309177), nearly half of the 50 PDAC patients had stable disease, 8 partially responded and one completely responded (Wainberg et al., 2020). In the second trial (NCT02331251), all patients achieved disease control, of which 3 showed partial response (Weiss et al., 2018). In a large phase II clinical study looking at the combination of gemcitabine and nab-paclitaxel with or without durvalumab and tremelimumab (NCT02879318), no benefits in terms of progression-free and overall survival were observed in the 191 metastatic PDAC patients; however, disease response rates were improved (Renouf et al., 2020).

Overall, chemoimmunotherapy improves response rates in PDAC compared to ICI or chemo monotherapies; however, these benefits remain limited and do not impact survival. Hence, the combination of ICI and chemotherapy is relevant in PDAC, but further potentiation of the immune system is needed to obtain significant benefit.

## PDAC Immune Microenvironment and IFN-I

PDAC is characterized by a dense tumor stroma mainly composed of the extracellular matrix, fibroblasts, and vasculature (Hosein et al., 2020). It also contains immunosuppressive cell types such as MDSCs and Tregs, which accumulate during disease progression to suppress CD4^+^ and CD8^+^ T-cell function, contributing to poor prognosis (Clark et al., 2007; Bayne et al., 2012; Thyagarajan et al., 2019; Huber et al., 2020).

Transcriptomic profiling of PDAC has identified 2 broad consensus subtypes, namely, classical and basal-like, which largely represent predominant neoplastic gene programs (Collisson et al., 2011; Moffitt et al., 2015; Bailey et al., 2016b). Additional transcriptomic subtypes, such as ADEX/exocrine-like and immunogenic, exhibit significant overlap with the classical subtype (Bailey et al., 2016b). Deconvolution of bulk transcriptomic RNAseq data using validated immune cell type signatures has demonstrated that the 2 consensus subtypes of PDAC are associated with distinct immune profiles (Bailey et al., 2016b; Karamitopoulou, 2019; Santiago et al., 2019). The basal-like subtype is usually associated with an “immune escaping” phenotype, with high Tregs and suppressive macrophages (M2 or m-MDSC) and low cytotoxic T cells (Wartenberg et al., 2018; Karamitopoulou, 2019). The classical subtype appears relatively “immune rich,” with lower Tregs and M2 macrophages and a higher percentage of CD4^+^ and CD8^+^ T cells as well as inflammatory (M1) macrophages (Wartenberg et al., 2018). IFN-I plays a pivotal role in regulating immunomodulatory chemokines and cytokines that in turn reshape the tumor immune microenvironment. In addition, IFN-I upregulates the expression of MHC-I and antigen presentation (Yang et al., 2004; Wan et al., 2012), increasing tumor cell recognition by the immune system. However, the presentation of neoantigens at the cell surface by MHC-I may be reduced by the targeted degradation of MHC-I in PDAC cells (Yamamoto et al., 2020). With respect to immune cells, IFN-I also enhances antigen presentation and cross-priming of the immune system as it stimulates DC activation and maturation (Lorenzi et al., 2011; Binnewies et al., 2019). In PDAC, DCs are rare and decrease upon disease progression, further limiting antigen cross-presentation and T-cell activity (Hiraoka et al., 2011). Similarly, IFN-I mediated activation of NK cell cytotoxicity (Swann et al., 2007; Bergamaschi et al., 2020) is impaired in PDAC (Marcon et al., 2020). Tumor-associated macrophages (TAMs) represent a dominant immune cell population in PDAC. High levels of the M2 or suppressive macrophage phenotype are associated with poor prognosis and disease progression (Kurahara et al., 2011; Candido et al., 2018). IFN-I can promote the antitumor M1 or inflammatory macrophage phenotype and enhance phagocytic functions (U'Ren et al., 2010; Sampson et al., 1991).

**FIGURE 2:**
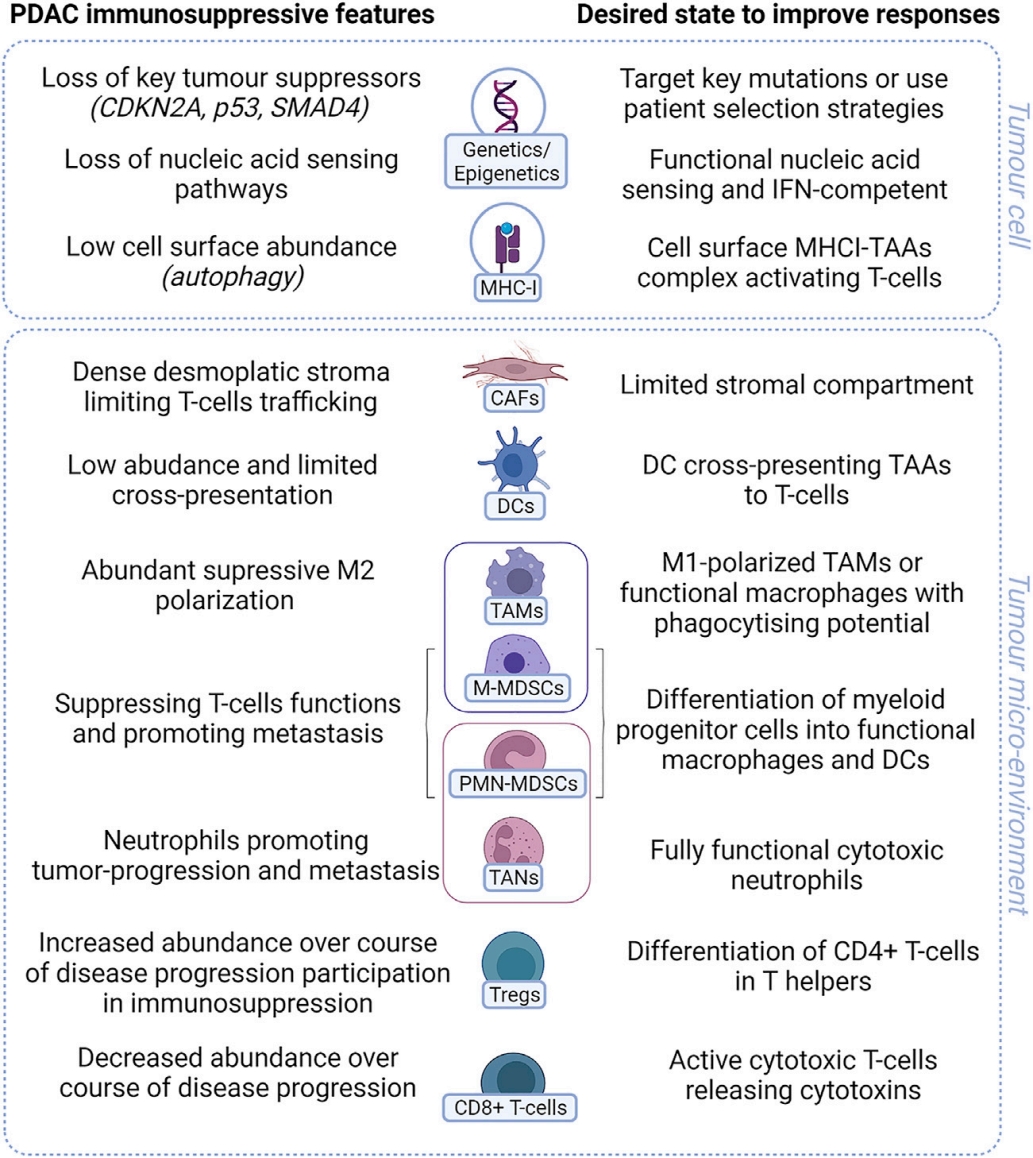
Key immunosuppressive features of PDAC. PDAC has many features that prevent activation of T cells in tumor. Both tumor cell intrinsic characteristics (genetic or changes in gene expression) along with tumor microenvironmental factors including suppressive or dysfunction cells all contribute to render PDAC immunologically “cold” with poor response to treatment. IFN= interferon. MHC-I= major histocompatibility complex class I; TAA= tumor-associated antigen; CAF= cancer-associated fibroblast; DC= dendritic cell; TAM= tumor-associated macrophage; M-MDSC= monocytic myeloid-derived suppressor cell; PMN-MDSC= polymorphonuclear myeloid-derived suppressor cell; TAN= tumor-associated neutrophil; Treg= regulatory T cells (Created using BioRender®).

The PDAC TME is accepted as being highly immunosuppressive (Figure 2). Many preclinical studies have shown the potential for TME modulators to enhance immunotherapy. Few have yet to translate to the clinic, but a number of agents targeting myeloid cells or stromal cells are being tested clinically with ICI or in chemotherapy/ICI combinations (Liu et al., 2019; Roma-Rodrigues et al., 2019; Ho et al., 2020; Zhong et al., 2020). CD4^+^ and CD8^+^ T cells are abundant at the initial stages of PDAC tumor development, and their presence and activation decrease during disease progression due to an immunosuppressive microenvironment (Knudsen et al., 2017). The dense fibroblastic stroma limits T-cell trafficking and access to the TME. MDSCs and Tregs suppress T-cell activation, which is already limited by poor antigen cross-presentation. Type I IFNs induce the secretion of cytokines such as CCL5, CXCL9, and CXCL10, which can attract T cells (Lorenzi et al., 2011) along with factors that negatively modulate immunosuppressive regulatory T cells (Gangaplara et al., 2018), facilitating effective cytotoxic T-cell functions. However, targeting different elements of the TME by modulation of myeloid cells with CXCR2, CCR2, or CSF1R (Steele et al., 2016; Candido et al., 2018; Nywening et al., 2018; Siolas et al., 2021) or CXCR4 antagonists (Feig et al., 2013; Biasci et al., 2020) can regulate the TME through changes in myeloid cells and stroma, improving preclinical responses to immune checkpoint inhibitors. Myeloid cells and the stroma can initiate IFN-I responses or be triggered as a result of tumor cell damage. The suppressive cell phenotypes in the TME may have limited capacity to enhance IFN-I or other innate signals. Targeting stromal cells in addition to tumor cells and T cells with chemotherapy and ICI may yield better clinical responses.

## Genetic Features of PDAC May Influence Immune Cell Function

PDAC tumors exhibit genetic characteristics that may underpin the lack of observed intrinsic responses to immune checkpoint therapy. Mutational heterogeneity is associated with a better response to ICI (Reuben et al., 2017; Iyer et al., 2021; Vitale et al., 2021). While PDAC tumors have many low-abundance mutations, there are four dominant driver mutations, namely, oncogenic activation of KRAS and inactivation of the tumor suppressors TP53, CDKN2A, and SMAD4 (Jones et al., 2008). CDKN2A loss correlates with reduced immune infiltrates in several tumor types, including PDAC (Siemers et al., 2017). Retrospective analysis of clinical data in different tumor types revealed an association of CDKN2A loss with worse overall and progression-free survival following ICI treatment in combination with chemotherapy (Horn et al., 2018; Gutiontov et al., 2021). Preclinically, specific loss of CDKN2A has also induced resistance to immunotherapy (Gutiontov et al., 2021; Han et al., 2021). CDKN2A is located on the human chromosome 9p21 locus (Sasaki et al., 2003), in close proximity to MTAP, JAK2, and a large cluster region coding for 16 IFN-I genes (Diaz, 1995). Co-deletion of these genes is common, for example, concomitant mutation of CDKN2A and MTAP in PDAC is estimated at 26% according to The Cancer Genome Atlas (TCGA) dataset (Mavrakis et al., 2016) and may also contribute to resistance to ICI-based therapy. In melanoma, CDKN2A-associated JAK2 mutation leads to loss of functional IFN-γ signaling which also limits response to immunotherapy (Horn et al., 2018). CDKN2A loss results in the activation of CDK4/6 kinases. In preclinical models, treatment with CDK4/6 inhibitors re-sensitized melanoma tumors (Jerby-Arnon et al., 2018) and syngeneic models (Deng et al., 2018) to treatment. In preclinical pancreatic cancer models, CDK4/6 inhibitors can influence tumor growth (Chou et al., 2018) and combine to sustain antitumor effects following chemotherapy (Salvador-Barbero et al., 2020). Combining the CDK4/6 inhibitor palbociclib and the MEK inhibitor trametinib not only reduced growth of patient-derived xenograft models but also enhanced response to PD-1 inhibition in a transplantable tumor model derived from the KPC model (Knudsen et al., 2021). This suggests that appropriate combinations with CDK4/6 inhibitors in PDAC may enhance immune response in tumors with loss of CDKN2A through tumor-centric and possibly TME mechanisms. In addition, it is not known whether loss of SMAD4 and p53 and mutation of KRAS also cooperate with CDKN2A/B loss to further increase the tumor immune-resistant status. For example, in lung cancer, loss of the tumor suppressor STK11 is regarded as driving resistance to both chemotherapy and immunotherapy (Skoulidis et al., 2018). Interestingly, it is the co-occurrence of a RAS mutation that renders these tumors most resistant to ICI-based treatments (Ricciuti et al., 2021). Therefore, given the dominant mutations in pancreatic cancer, it may be challenging to achieve strong immune activation.

The IFN-I genes are also expressed on Chr9 (close to CDKN2A and MTAP) and can sometimes be lost. It is also possible that even when not deleted, the disruption of other genes in the locus may reduce gene expression. Tumor cells with reduced IFN-I expression may evade immune cells due to a lack of IFN-α and -*ß* cross talk to the immune system. Loss or reduction of this central coordinator mechanism for the tumor–immune interaction would contribute to immune escape and ICI resistance (Grard et al., 2021), especially in the context of tumor-targeted combination approaches.

Less than 4% of PDAC tumors comprise mutations in DNA damage repair genes, such as BRCA1, BRCA2, or PALB2 (Waddell et al., 2015; Bailey et al., 2016b). BRCA1- and BRCA2-deficient tumors are associated with increased immune infiltrates in some patients; however, rates of response to ICI are low (Sønderstrup et al., 2019; Wen and Leong, 2019; Mei et al., 2020). Recent evidence in mouse models of breast and colorectal cancers suggests that BRCA2-deficient tumors are more susceptible to ICI than BRCA1-deficient tumors (Samstein et al., 2020; Zhou and Li, 2021). ARID1A alteration is found in about 6% of PDAC patients (Cancer Genome Atlas Resea, 2017) and has been observed to modulate responses to immunotherapy. In fact, ARID1A deficiency has been shown to lead to the inactivation of MMR genes, increase in PD-L1 expression, increase in tumor mutation burden through DDR dysfunction *via* ATR inhibition, and TIL recruitment and inflammatory response by IL-6 production (Hu et al., 2020; Wang et al., 2020). Consistently, ARID1A-mutated patients showed better response to ICI with prolonged progression-free and overall survival observed in various solid cancer types, suggesting that ARID1A deficiency may be a patient selection biomarker for ICI combinations (Jiang et al., 2020; Okamura et al., 2020). It is unlikely that mutation status alone will be sufficient to drive patient segmentation in PDAC; therefore, new strategies to define patient subsets for specific combination treatments will be important.

## Triggering Innate Immune Responses in PDAC

To maintain immunological homeostasis, an organism must discriminate self from non-self. Upon infection, viral nucleic acids stimulate antiviral responses, but the same innate pathways can also recognize damaged self-DNA and/or RNA (Iurescia et al., 2018). Activation or dysfunctional regulation of proteins involved in these pathways causes disorders known as interferonopathies, which are associated with increased interferon production. For example, the Mendelian autoinflammatory disorder Aicardi–Goutieres syndrome is a type I interferonopathy caused by mutations in genes such as TREX1, which plays a pivotal role in endogenous nucleic acid sensing (Tao et al., 2019; Baris et al., 2021).

Chromosome instability (CIN) is a hallmark of cancer (Pikor et al., 2013), and damaged DNA within micronuclei has been demonstrated to induce innate immune responses (Mackenzie et al., 2017). Although nuclear DNA is shielded from cytoplasmic nucleic acid sensors by the nuclear membrane, membrane rupture during mitosis and/or cytotoxic stress can expose nucleic acids to pattern recognition receptors (PRRs). PRR stimulation activates the immune system through the modulation of the tumor cell surface and secreted proteins (Maciejowski and Hatch, 2020). PDAC is characterized by DDR deficiency, CIN, and metabolic stress (Bailey et al., 2016b; Aguirre et al., 2018). These tumor cell intrinsic properties should trigger canonical innate immune pathways. However, responses are limited in PDAC because of the activation of anti-autoimmune regulatory mechanisms that facilitate immune evasion and disease progression (Moskovitz et al., 2003; Waddell et al., 2015; Bailey et al., 2016b).

## DNA- and RNA-Sensing Signaling Pathways in PDAC

**FIGURE 3:**
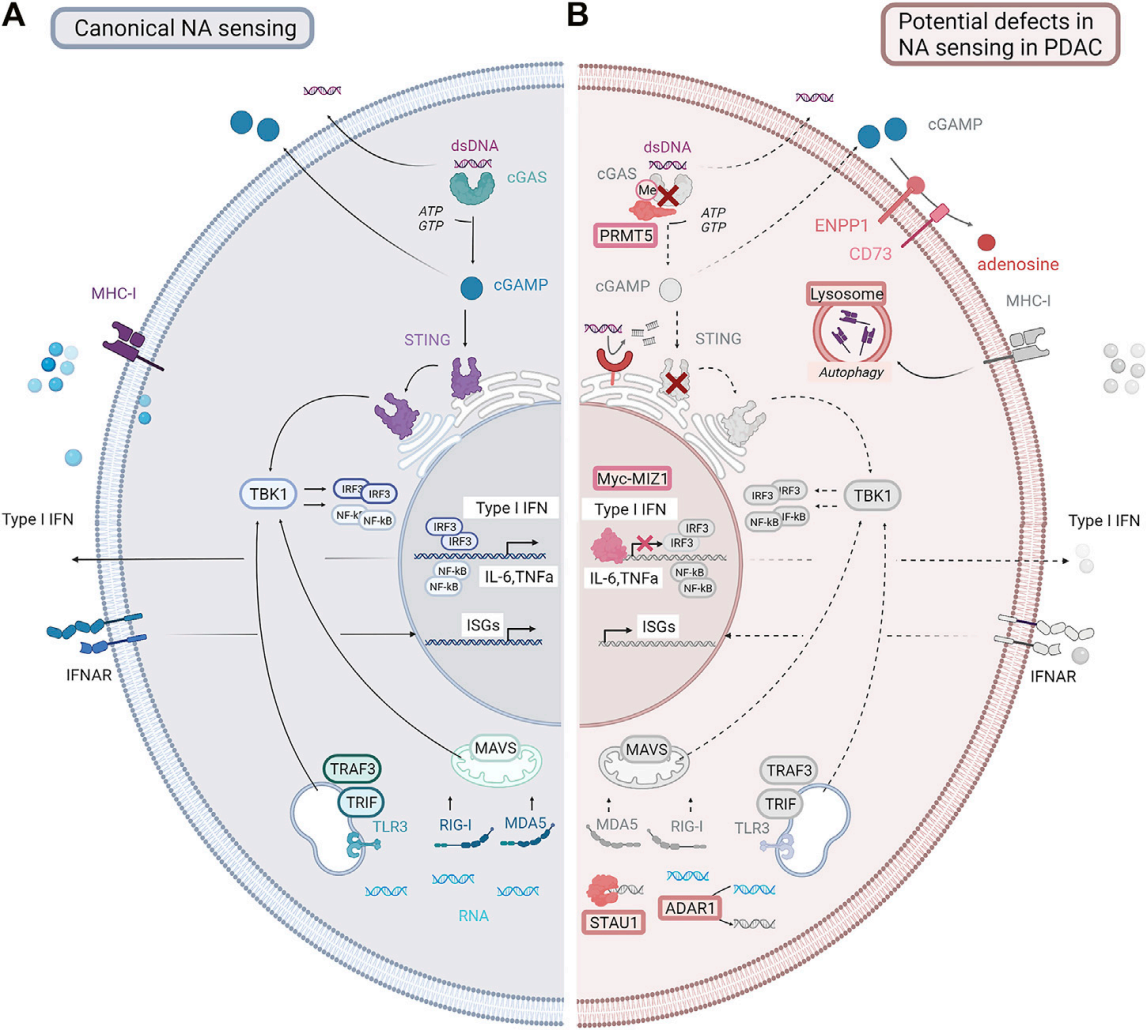
Canonical nucleic acid-sensing pathways and potential defects in PDAC. **(A)** Canonical nucleic acid-sensing pathways detect dsDNA or dsRNA fragments (DAMPs) generated as a result of stress, or following drug treatment, which accumulate in the cytoplasm. dsDNA fragments activate the cGAS-STING pathway. dsRNA fragments activate the MAVS/RIG-I/MDA5 or other sensing pathways, for example, TRAF3/TRIF. These complexes drive TBK1, resulting in endogenous type I interferon production and interferon-stimulated gene expression. Nucleic acids along with signaling molecules such as cGAMP can be secreted by tumor cells and activate nucleic acid-sensing or innate damage-sensing pathways in normal immune or stromal cells. Secreted IFN-I can activate IFN signaling in other tumor cells. Antigen presentation is upregulated with increased MHC-I expression. **(B)** In PDAC, these pathways are disrupted. The cGAS-STING pathway can be lost by deletion or downregulation. RNA sensing can be inhibited through the upregulation of STAU and ADAR1. cGAMP can be degraded by the upregulation of ENPP1. IFNAR receptor can be downregulated. Antigen presentation can be inhibited by the downregulation of MHC1 expression. *Pathways that are lost or downregulated in PDAC are shown in gray*. NA= nucleic acid; dsDNA= double-stranded DNA; cGAMP = cyclic guanosine monophosphate; IFN = interferon; ISGs = interferon stimulated genes; IFNAR: interferon α/β receptor; MHC-I = major histocompatibility complex class I (Created using BioRender®).

Chemotherapy, radiation, and therapeutics that damage DNA can prime immune responses through the stimulation of nucleic acid-sensing pathways. DNA or RNA fragments stimulate innate immune pathways in the tumor, including the secretion of IFN-I, which cross talks to immune cells (Figure 3). Double-stranded DNA (dsDNA) is generally located in the nucleus. However, in cells with CIN, due to DNA damage and/or defects in DNA damage repair machinery, cell cycle regulation (Fenech et al., 2011; Crasta et al., 2012; Sahin et al., 2016), or drug treatment dsDNA is found in the cytoplasm. Canonically, cytoplasmic dsDNA is sensed by cGAS (cyclic GMP-AMP synthase), a DNA-binding protein that catalyzes the production of the second messenger cGAMP (2′-3′ cyclic GMP-AMP) (Li et al., 2013). cGAMP interacts with the adaptor protein stimulator of interferon genes (STING) (Ablasser et al., 2013; Kato et al., 2017), causing dimerization and translocation from the endoplasmic reticulum to the Golgi (Ishikawa and Barber, 2008; Ishikawa et al., 2009). STING activates the kinase TBK1, driving translocation of the transcription factor IRF3 to the nucleus and inducing the expression of IFN-I (Liu et al., 2015). This sensing pathway can also be triggered in non-tumor cells with build-up of cytoplasmic dsDNA in the TME, with a transfer of dsDNA and/or cGAMP from tumor to host cells (Schadt et al., 2019; Zou et al., 2021). STING and TBK1 activation trigger the recruitment of IκB kinases (IKK), which results in NF-κB pathway activation by phosphorylation of the inhibitory IκB, thus enabling the translocation of NF-κB transcription factors to the nucleus (Abe and Barber, 2014; Yum et al., 2021). Similarly, the NF-κB and IFN-I pathways can be activated by sensing DNA through endosomal TLR3 and TLR9 (Kawai and Akira, 2007). The STING-TBK1 signaling axis has shown functionality in several PDAC models upon stimulation with STING agonists or modulators (Jing et al., 2019; Ren et al., 2020; Liang et al., 2021; Miller et al., 2021). However, it is not clear whether STING responses are functional in the majority of PDAC tumors, or whether the pathway is attenuated as tumors progress. Interestingly, cGAS-STING pathway agonists show efficacy in preclinical PDAC tumors by targeting macrophages in the TME (Ager et al., 2021), suggesting that tumor cells or cells within the TME may respond to activation of this pathway.

RNA sensing allows cells to detect cytoplasmic double-stranded RNAs and specific single-stranded RNAs, which are usually signs of viral infection. However, following treatment (Ranoa et al., 2016), cytoplasmic dsRNAs may accumulate in treated cells and be recognized by RNA-sensing pathways. Cytoplasmic dsRNAs are sensed, according to their size and location, by toll-like receptors such as TLR3 located in endosomes (Alexopoulou et al., 2001) or cytosolic ubiquitously expressed RIG-I-like receptors (RLRs) such as RIG-I and melanoma differentiation associated gene-5 (MDA5) (Hornung et al., 2006; Kato et al., 2006). The former signals through the adaptor protein TRIF, while the latter induces the activation of mitochondrial antiviral signaling protein (MAVS). Following a cascade of downstream events, both converged on TBK1, and subsequent IRF3 activation results in IFN-I and NF-kB responses (Seth et al., 2005; Sun et al., 2006; Kawai and Akira, 2018). These pathways are functional in preclinical models of PDAC following stimulation by PRR agonists (Duewell et al., 2014; Metzger et al., 2019). It is therefore possible that triggering these pathways with radiation or chemotherapy, or directly with pathway agonists to target STING or RIG-I in both tumor and the TME could add benefit as part of a combination strategy with ICI.

## Mechanisms Regulating DNA- and RNA-Induced IFN Responses in PDAC

As cancer cells are altered-self cells, recognition by the immune system can be facilitated by damage-associated molecular patterns (DAMPs). The term DAMP encompasses a group of molecules that can signal in a cell-intrinsic manner or trigger cross talk to the innate immune system after being released from damaged cells (e.g., cGAMP, DNA or RNA fragments, or intracellular proteins such as actin, HMGB1, and histones) (reviewed in depth in Gong et al. (2020)). DAMPs commonly activate a range of PRRs and trigger IFN-I responses (Teijaro, 2016; Musella et al., 2017) as a result of genetic changes or drug treatments (Stetson and Medzhitov, 2006; Reikine et al., 2014). Tumor cells commonly undergo changes that abrogate or make these sensing pathways less effective or reduce the secretion of DAMPs.

TREX1 and ENPP1 can antagonize signals resulting from damaged DNA (Figure 3). Both enzymes are highly expressed in PDAC (Carozza et al., 2020), and overexpression reduces the activation of the cGAS-STING pathway. TREX1 is an ER-associated exonuclease that degrades cytosolic dsDNA and therefore restrains cGAS-STING activation (Mohr et al., 2021). ENPP1 is a transmembrane protein with a hydrolase extracellular domain capable of cleaving a variety of substrates including cGAMP preventing activation of the cGAS-STING pathway in surrounding cells (Li et al., 2021). In preclinical studies, cGAMP plays a role in driving cross talk to the TME following radiation treatment (Vijayan et al., 2017). Moreover, cGAMP hydrolysis by ENPP1 results in the formation of AMP, a substrate of NT5E, a transmembrane hydrolase that catalyzes the formation of adenosine (Allard et al., 2019) which also drives immunosuppression in the TME (Stagg et al., 2010; Vijayan et al., 2017). Whether cGAMP and ENPP1 play a similar role in PDAC has not been explored in detail.

Epigenetic regulation is another strategy cancer cells use to modulate response to DAMPs and attenuate PRR activation or downstream signaling. cGAS and STING are epigenetically silenced by the methylation of their respective promoter regions in PDAC (Konno et al., 2018). KRAS and MYC aberrations, which are among the most common in PDAC, suppress IFN-I response by inducing binding of the Myc–MIZ1 complex to IFN regulator promoters (IRF5, IRF7, STAT1, and STAT2) (Muthalagu et al., 2020). Posttranslational modifications such as methylation, phosphorylation, sumoylation, acetylation, and ubiquitination also modulate constituents of the cGAS-STING pathway in different cancer types (Brown et al., 2018; Tan et al., 2018; Wu et al., 2019; Zhang et al., 2020; Ma et al., 2021). Interestingly, the epigenetic factor PRMT5, which is upregulated in PDAC and correlates with poorer survival (Qin et al., 2019), directly methylates cGAS and limits binding to dsDNA (Muthalagu et al., 2020). It is possible that inhibiting TREX1 or ENPP1 function could result in more effective activation of DNA-sensing pathways in specific subsets of PDAC (e.g., subsets with defects in DNA repair pathways), or following treatment with chemotherapy or radiation.

RNA-sensing pathways can also initiate damage signals, and proteins that prevent sensing of dsRNA can be upregulated in tumors (Figure 3). For example, the protein STAU1 stabilizes dsRNA, preventing recognition by RNA-sensing proteins (Chengjin et al., 2018), while ADAR1 targets modified RNA fragments, assisting evasion from triggering of RNA-sensing complexes (Mehdipour et al., 2020). ADAR1 destabilizes specific inverted Alu repeats in dsRNA motifs by catalyzing adenosine-to-inosine editing. Alu repeats are a major source of drug-induced dsRNA, and their destabilization by ADAR1 limits activation of MDA5 and RIG-I (Pichlmair and Reis e Sousa, 2007; Liddicoat et al., 2015; Wang et al., 2017; Herbert, 2019). While the regulation of the RNA-sensing machinery has not been widely explored in PDAC, ADAR1 is upregulated in PDAC and is associated with poor prognosis (Sun et al., 2020). Interestingly, IFN-I also induces tumor cell apoptosis and DAMP release in cell lines (Kimura et al., 2003; Gómez-Benito et al., 2007; Kazaana et al., 2019), including PDAC cells (Vitale et al., 2007) (Figure 1).

The potential to harness these pathways to initiate an immune trigger in PDAC is underexplored. Chemotherapy may trigger responses in the tumor cells or the TME, while fragments released from the tumor may stimulate the TME. Given the high density of macrophage-like cells found in PDAC and the high stromal content, agonists of DNA- or RNA-sensing pathways could also play an important role in enhancing more sustained immune responses in the appropriate treatment regimens.

## Therapeutic Strategies to Restore Tumor IFN-I or Innate Responses in PDAC to Maximize Immune Cell Engagement

There are a variety of pathways that can be activated or inhibited to elicit an interferon response. Reducing the presence of DAMPs is a strategy for cancer cells to evade NA sensing; therefore, increasing DAMP expression could initiate an effective IFN-I response. This could be achieved by targeting the DNA damage response. Conventional treatments such as chemo- and radiotherapy act as potent DNA-damaging agents that can induce IFN-I and recruit antigen-presenting cells (Lugade et al., 2005). PARP inhibition leads to the accumulation of cytosolic DNA by blocking DNA repair. Recently, the PARP inhibitor (PARPi) olaparib has been approved by the FDA for the treatment of BRCA-mutated metastatic PDAC after having shown promising results in the POLO clinical trial (Golan et al., 2019). Interestingly, phase II and III clinical trials have shown PARPi efficacy in patients with non-mutated BRCA1/2 ovarian cancer, suggesting a potential use in a broader spectrum of patients (Gelmon et al., 2011; Mirza et al., 2016). In subcutaneous mouse syngeneic tumor models, the combination of PARPi and anti-PD-L1 treatment increases therapeutic efficacy in BRCA-deficient tumors (Shen et al., 2019). In PDAC tumors with BRCA mutation, inhibiting PARP in combination with ICI may give further enhanced benefits. This same principle could apply to other PDAC subtypes with deficient DNA repair pathways, perhaps giving similar but more sustained IFN-I and innate stimulation than that achieved with chemotherapy.

Inhibiting the function of ATM which repairs double-stranded breaks also increases cytoplasmic DNA (Zhang et al., 2019), TBK1 phosphorylation, and IFN-I expression in preclinical PDAC models. Interestingly, ATM loss of function (which renders cells dependent on the alternative DNA repair enzyme ATR) has been associated with an immune-rich phenotype in PDAC (Wartenberg et al., 2018). Preclinically inhibiting ATR alone or in combination with PARP inhibition has shown therapeutic benefit (Dunlop et al., 2020; Dreyer et al., 2021; Gout et al., 2021; Parsels et al., 2021). Finally, inhibiting DNA-PK, which facilitates non-homologous end joining in combination with radiotherapy increases micronuclei formation and DNA damage, activates IFN-I and increases CD8^+^ T-cell response (Ciszewski et al., 2014). Therefore, in specific patient subgroups, PARP, ATM, ATR, or DNA-PK inhibitors given chronically (alone or in combination) may sustain DNA damage to trigger immune engagement. Indeed targeting these enzymes with more chronic treatment may be more effective at driving IFN-I and other damage responses than chemotherapy, which only achieve short transient stimulation of these pathways (Reisländer et al., 2020).

There are other ways to increase nucleic acid-mediated activation of DNA damage or stress pathways. Although not investigated in PDAC, the nucleoside analog 6-thio-DG, which induces DNA damage and telomerase stress, activates STING, IFN-I signaling, and CD8^+^ T cells in colorectal cancer models (Mender et al., 2020). The DNA-sensing cGAS-STING pathway can also be targeted directly with agonists. Intratumoral injection of the STING agonist ADU-S100 combined with radiotherapy increased tumor interferon-stimulated gene expression and T-cell infiltration, causing a reduction in local and distal tumor burden in PDAC murine models (Vonderhaar et al., 2021). Combining STING agonists with anti-PD-1 also showed promising antitumor activity in preclinical mouse syngeneic models (Ghaffari et al., 2018; Kim et al., 2019; Lemos et al., 2020). STING activation in PDAC can occur within either tumor or macrophages to drive a response. Despite these promising preclinical studies, selectively triggering STING also presents challenges. Overactivation of STING may also drive systemic toxicity through the release of TNFα and other cytokines and may limit the activation that can be achieved, or the duration of treatment. Preclinical tool compounds capable of stimulating the RIG-I RNA-sensing pathway have also shown interesting proof of concept in PDAC models (Ellermeier et al., 2013; Bhoopathi et al., 2014; Das et al., 2019) and may provide an alternate approach in tumors where the cGAS-STING pathway is not functional.

Activating PRRs or reducing suppression by inhibiting negative regulators will also drive IFN-I induction. Although not explored extensively in PDAC, administration of TLR7/8 agonists such as R848 has shown promising results in preclinical models (Michaelis et al., 2019). However, as with STING agonists, these agents can be limited by toxicity, requiring careful scheduling of treatment or the requirement for intratumoral injection to mitigate toxicity (Mullins et al., 2019). TLR9 agonists SD-10-1 and CMP0001 sensitized ICI-resistant mouse models of the colon (Wang et al., 2016) and head and neck squamous cell carcinoma (Sato-Kaneko et al., 2017) to PD-1 blockade. Response was linked to IFN-I stimulation, increase in interferon-stimulated gene expression, and subsequently expansion of CD8^+^ T cells. Approaches to stimulate these pathways may be worth considering for PDAC to enhance the triggering of immune responses.

Modulators of the DNA methylation state can cause DNA or RNA stress within cells. DNA methyltransferase inhibitors (DNMTi) reduce methylation of endogenous retroelements (Roulois et al., 2015) and increase levels of cytosolic dsRNA (Goel et al., 2017). 5-Azacytidine (a DNMTi) induced re-expression of silenced genes leading to increased T-cell-stimulating chemokines *in vitro* and increased T-cell infiltration in PDAC *in vivo* models (Ebelt et al., 2020). Effects of epigenetic modulators can however be context-dependent. The inhibition of PRMT5 with EPZ015666 increased IFN-I and cGAMP production upon stimulation with DNA fragments *in vitro* (Ma et al., 2021). However, in another study, EPZ015666 decreased IFN-I expression and impaired interferon-stimulated gene expression upon stimulation with poly(I:C) (Cui et al., 2020). Last, consistent with ADAR1 being associated with dampening RNA-sensing pathways, knockout in tumor cells conferred vulnerability to immune checkpoint inhibitors (Ishizuka et al., 2019).

Vaccines or oncolytic viruses can stimulate IFN-I responses, while delivery of recombinant IFN-α or -β to tumors agonizes the pathway directly. Early trials with IFN-I conjugates were not successful, largely due to toxicity issues; however, next-generation approaches that deliver IFN-I conjugates more safely are being developed. Tumor vaccines will stimulate broad innate immune responses. Both cell-based tumor vaccine approaches such as GVAX (Wu et al., 2020b) and GVAX, and a *Listeria*-based vaccine expressing mesothelin (an antigen upregulated in pancreatic cancer) have been trialed (Le et al., 2019). Unfortunately, these vaccine-based approaches have not yielded positive clinical signals. Oncolytic viruses can stimulate significant IFN-I induction (reviewed in Harrington et al. (2019); Evgin et al. (2020); Cao et al. (2021); Rosewell Shaw et al. (2021)) and drive sustained T-cell activation. Novel strategies combining oncolytic viruses with CAR-T therapy are being developed to drive targeted sustained responses in preclinical pancreatic cancer tumor models.

There are many different strategies to increase tumor immune cross talk through IFN-I responses. However, these approaches are challenging, and therapeutic index is likely to be an issue requiring careful dose selection clinically and the ability to focus on specific patient subsets to induce an effective innate immune response (Konno et al., 2018) (Salvador-Barbero et al., 2020) (Muthalagu et al., 2020). Moreover, IFN-I may act differently when induced acutely or with sustained chronic upregulation. How the IFN-I response is modulated and for how long needs to be considered as complicated regulatory mechanisms can have both activating and suppressive effects, depending on the context.

## Conclusion

PDAC has limited sensitivity to chemotherapy and resistance to most current immunotherapy approaches. While chemoimmunotherapy shows better overall responses than chemotherapy or immunotherapy alone, this benefit is marginal for most patients. In PDAC, many mechanisms may prevent the activation of the immune response, from failure to sustain tumor damage- and stress-related immune triggers (e.g., IFN-I release, antigen presentation, or DAMP release) to indirect resistance in the TME. IFN responses can however be stimulated with tumor-targeted treatment (e.g., chemotherapy and DDR inhibitors) or induced in the TME. Improved clinical responses could be achieved by enhancing the cross talk between the tumor and TME through the induction of IFN-I signaling, and there are promising approaches to achieve this in the right setting. A number of clinical translational programs such as Pan Can, ESPAC, and Precision Panc are using multi-omic assessments of human clinical trial samples to look at features that influence response to treatment. Studies such as these can provide new ways to look at where and how IFN response may be suppressed in patients as well as identify tumors that may respond well to specific treatment strategies. This work will contribute to our understanding of the intrinsic tumor cell-initiated immunomodulatory pathways in PDAC. Moreover, focusing on treating subsets of tumors in a biomarker and mechanism linked way will hopefully enable the field to identify and build on efficacy signals with more confidence. Ultimately, this will guide the development of improved combination strategies that potentiate immune response in this challenging disease.
